# Vascular smooth muscle cell senescence accelerates medin aggregation via small extracellular vesicle secretion and extracellular matrix reorganization

**DOI:** 10.1111/acel.13746

**Published:** 2022-11-25

**Authors:** Meredith Whitehead, Syabira Yusoff, Sadia Ahmad, Lukas Schmidt, Manuel Mayr, Jillian Madine, David Middleton, Catherine M. Shanahan

**Affiliations:** ^1^ School of Cardiovascular and Metabolic Medicine & Sciences King's College London London UK; ^2^ Institute of Systems, Molecular and Integrative Biology University of Liverpool London UK; ^3^ Department of Chemistry Lancaster University Lancaster UK

**Keywords:** amyloid, extracellular matrix, extracellular vesicles, proteoglycans

## Abstract

Vascular amyloidosis, caused when peptide monomers aggregate into insoluble amyloid, is a prevalent age‐associated pathology. Aortic medial amyloid (AMA) is the most common human amyloid and is composed of medin, a 50‐amino acid peptide. Emerging evidence has implicated extracellular vesicles (EVs) as mediators of pathological amyloid accumulation in the extracellular matrix (ECM). To determine the mechanisms of AMA formation with age, we explored the impact of vascular smooth muscle cell (VSMC) senescence, EV secretion, and ECM remodeling on medin accumulation. Medin was detected in EVs secreted from primary VSMCs. Small, round medin aggregates colocalized with EV markers in decellularized ECM in vitro and medin was shown on the surface of EVs deposited in the ECM. Decreasing EV secretion with an inhibitor attenuated aggregation and deposition of medin in the ECM. Medin accumulation in the aortic wall of human subjects was strongly correlated with age and VSMC senescence increased EV secretion, increased EV medin loading and triggered deposition of fibril‐like medin. Proteomic analysis showed VSMC senescence induced changes in EV cargo and ECM composition, which led to enhanced EV‐ECM binding and accelerated medin aggregation. Abundance of the proteoglycan, HSPG2, was increased in the senescent ECM and colocalized with EVs and medin. Isolated EVs selectively bound to HSPG2 in the ECM and its knock‐down decreased formation of fibril‐like medin structures. These data identify VSMC‐derived EVs and HSPG2 in the ECM as key mediators of medin accumulation, contributing to age‐associated AMA development.

## Graphical abstract

This study has elucidated how during ageing, VSMCs regulate medin accumulation via secretion of sEVs, changes in sEV cargoes and ECM composition. We showed that VSMC senescence increased secretion of sEVs and the accumulation of medin in the ECM by enhancing medin aggregation on the EV surface and enhancing EV binding to HSPG2 in the ECM. In addition, increased expression and deposition of HSPG2 in the aged ECM triggered formation of fibril‐like medin, further enhancing the development of AMA.

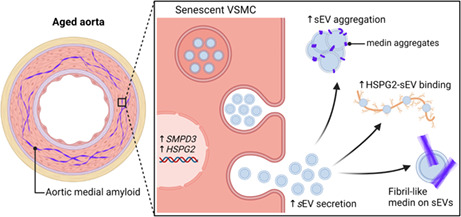

## 1 INTRODUCTION

Vascular amyloidosis, caused when soluble protein or peptide monomers aggregate into insoluble amyloid, is a prevalent age‐associated pathology. However, the mechanisms regulating amyloid formation as well as its pathological impact on vessel function remain poorly understood. Aortic medial amyloid (AMA) is the most common human amyloid and present in the vessel wall of 97% of Caucasians aged over 50 years (Häggqvist et al., 1999). AMA is composed of medin, a 50‐amino acid fragment cleaved from a secreted glycoprotein, milk fat globule EGF factor 8 (MFG‐E8). MFG‐E8 is expressed by vascular smooth muscle cells (VSMCs) and its deposition in the vessel wall increases with age and injury (Cheng et al., 2012; Miura et al., 2019). It is a multi‐functional protein composed of several domains, including an RGD peptide that can bind integrins and two C2‐like domains which bind phospholipids. It has been shown to regulate opsonization through phosphatidylserine binding on apoptotic cells and integrins on macrophages (Fricker et al., 2012). Medin is composed of a fragment from within the membrane‐binding C2‐like domain of MFG‐E8 and is therefore able to interact with phospholipid membranes. Both MFG‐E8 and medin can also bind elastin, accounting for its localization in large vessels, and it has been suggested that elastic fibers may act as a nucleation site for the formation of medin amyloid (Peng et al., 2002). However, despite the prevalence of medin in the vasculature, the mechanism of MFG‐E8 cleavage to form medin is currently unknown and it remains unclear how it accumulates with age and whether other ECM components or processes are involved.

Emerging evidence has implicated extracellular vesicles (EVs), including exosomes and multi‐vesicular endosomes (MVEs), as mediators of physiological (van Niel et al., 2011) and age‐related, pathological amyloid metabolism (Bellingham et al., 2012). EVs have been shown to enhance amyloid formation by acting as a nucleation site for aggregation and can contribute to the propagation of amyloid through uptake by neighboring cells (Aguzzi & Rajendran, 2009). For example, exosomes play a pivotal role in aggregation of premelanosomal protein (PMEL) in non‐pathological amyloid formation in the skin and eye, by acting as a scaffold for melanin polymerization (van Niel et al., 2011). Cleavage of PMEL into amyloidogenic fragments occurs in MVEs and their aggregation is nucleated by intraluminal vesicles, which are formed in MVEs prior to secretion as exosomes. A similar mechanism is observed during accumulation of β‐amyloid (Aβ) in the brain in Alzheimer's disease (AD). Processing of amyloid precursor protein (APP) occurs predominantly in the endosomal pathway and Aβ is then secreted by exosomes into the extracellular space, where they function as a nucleation site for amyloid plaque formation (Rajendran et al., 2006, 2007). Importantly, proteomics studies have identified MFG‐E8 as an EV cargo in a number of cell types, including VSMCs (Kapustin et al., 2015), and the ability of both MFG‐E8 and medin to bind to lipid membranes raises the possibility exosomes could be involved in extracellular medin accumulation; however, to date no studies have examined this pathway.

A hallmark of vascular ageing is the accumulation of senescent VSMCs, characterized by cell cycle arrest, DNA damage, and the secretion of inflammatory cytokines, growth factors and proteases (Acosta et al., 2013; Coppe et al., 2010). Extensive research has implicated VSMC senescence in the development of age‐associated vascular pathologies, such as calcification, hypertension, and atherosclerosis (Chi et al., 2019). However, there has been no investigation into the role of VSMC senescence in vascular amyloidosis. Therefore, to determine the mechanisms of AMA formation, we explored the impact of VSMC senescence, EV secretion and ECM remodeling on medin accumulation. We show that VSMC senescence is associated with increased EV secretion and proteoglycan deposition which synergistically act to accelerate medin amyloid formation.

## 2 RESULTS

### 2.1 Extracellular medin deposition and aggregation is mediated by VSMC EVs

**FIGURE 1:**
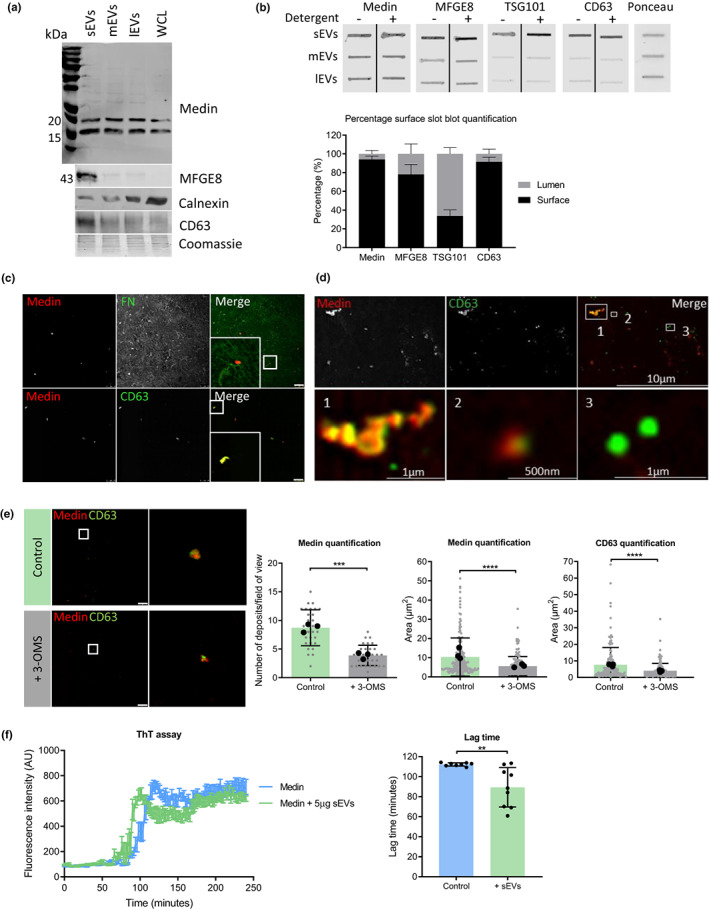
Extracellular medin deposition and aggregation is mediated by VSMC EVs. (a) Western blot of VSMC small (sEVs), medium (mEVs), large (lEVs) extracellular vesicles (EVs) and whole cell lysate (WCL). CD63 was used as a sEV marker and calnexin was used as a cell marker. (b) Slot blot of EVs and quantification showing surface localization of medin and MFG‐E8. TSG101 was used as a control which localises mainly inside the lumen of EVs, while CD63 was used as a surface protein control (*n* = 3 from 35‐year‐old female (35F)). (c) Immunofluorescence for medin and CD63 in decellularized ECM. Scale bar 25 μm. (d) Super‐resolution microscopy of (1) larger medin and EV aggregates, (2) individual sEVs with medin on the surface and (3) individual sEVs which do not colocalize with medin. (e) Immunofluorescent staining and quantification of ECM synthesized with or without EV secretion inhibitor 3‐O‐methylsphingomyelin (3‐OMS). Grey data points represent deposits within a field of view and black points represent average from each donor. Scale bar 25 μm, (*n* = 6 from 35F, 22‐year‐old male (22), 20‐year‐old male (20 M)). Unpaired Student's t test, ***p < 0.005, ****p < 0.001. (f) Thioflavin T (ThT) aggregation assay and quantification of recombinant medin peptide with or without sEVs added (*n* = 8–9 from 35F). Unpaired Student's t test, **p < 0.01. All data is displayed as mean ± SD and was tested for normality using Shapiro–Wilk test.

To determine whether EVs are involved in the secretion and deposition of medin, EVs were isolated from primary, human aortic VSMCs by differential ultracentrifugation and analyzed by Western blotting. MFG‐E8 was highly enriched in the small EV (sEV) fraction, and detectable in medium EVs (mEVs) and large EVs (lEVs). Medin was detectable as 16 kDa and 20 kDa bands, potentially corresponding to peptide oligomers, in all three EV fractions and cell lysates (Figure 1a). These bands also correspond to the size observed in U2OS cells. There were endogenous medin bands detected in the un‐transfected cells, and these were increased in cells overexpressing only the medin fragment of MFG‐E8 (Figure S1a). The medin antibody used (18G1 clone) specifically detects medin, by binding to a neo‐epitope which only becomes accessible following cleavage of medin from MFG‐E8. Validation was performed by using a different medin antibody (6B3 clone) which can detect medin and MFG‐E8. Western blotting showed bands at 16 kDa and 20 kDa, corresponding to medin, and a band at around 43 kDa in the sEV fraction, corresponding to MFG‐E8 (Figure S1b). In addition, knockdown of MFG‐E8 with siRNA in VSMCs (Figure S1c) decreased the 16 kDa and 20 kDa bands, providing further validation of the medin antibody (Figure S1d).

To distinguish between surface and lumen bound proteins, EVs were permeabilized by Tween‐20 or blotted in the absence of Tween‐20 to only detect surface proteins. TSG101, which is located predominantly inside exosomes, and CD63 were used as controls to show detection in the lumen and on the surface, respectively. TSG101 was detected in higher amounts in Tween‐20‐containing conditions, indicating it is localized more in the lumen of sEVs (Figure 1b). Conversely, CD63 was detected in equal amounts in both conditions, confirming its surface localization. MFG‐E8 was mostly located on the surface of sEVs, with quantification showing 79% surface bound. Medin was detected at similar levels in Tween‐20‐free and Tween‐20 containing conditions, and quantification of the percentage of surface‐bound protein showed 97% of medin was located on the sEV surface. To further validate the presence of medin in sEVs, we used ExoView (NanoView Biosciences). Medin was detected on the surface of sEVs captured on a chip by CD63 and CD9 antibodies (Figure S1e). The number of particles bound to the chips was quantified and showed more medin positive sEVs bound to CD63 and CD9 antibody‐coated chips, compared with the IgG control.

Next, we developed an in vitro model of decellularized, VSMC‐derived ECM to visualize the deposition of medin in the ECM. Immunofluorescent staining (IF) for fibronectin showed VSMCs deposit an intact ECM (Figure S1f) and absence of DAPI staining following cell lysis confirmed the successful removal of VMSCs (Figure S1g). The medin aggregates were present in the ECM prior to VSMC removal, indicating they were deposited by VSMCs and not an artefact of cell lysis (Figure S1h). Medin was detected in the ECM in a punctate pattern co‐localizing with the previously identified sEV markers CD63, fetuin A and annexin A6 (Figure 1c and Figure S1i) (Kapustin et al., 2015; Kapustin & Shanahan, 2016; Rentero et al., 2018; Zhou et al., 2006). IF showed medin present inside VSMCs, with some colocalization with CD63, suggesting medin is secreted from inside the cell and not binding to sEVs extracellularly (Figure S1j). Medin was also detected in the ECM with the 6B3 antibody, while there was no MFG‐E8 detected with an MFG‐E8‐specific antibody, indicating there is deposition medin and not MFG‐E8 in the ECM (Figure S1k,l). Super‐resolution microscopy confirmed the colocalization of medin and CD63‐positive sEVs, which were approximately 200 nm in diameter (Figure 1d). Medin appeared to be on the surface of sEVs (inset 2 of Figure 1d) with the red staining surrounding a core of CD63. Larger aggregates of CD63‐positive sEVs approximately 10μm^2^ were also observed colocalizing with medin.

To substantiate the role of sEVs in extracellular medin deposition, VSMCs were treated with an EV secretion inhibitor, 3‐O‐methylsphingomyelin (3‐OMS), during ECM synthesis. A CD63‐beads assay and flow cytometry analysis confirmed 3‐OMS significantly decreased secretion of CD63 and CD81‐positive EVs, while not affecting cell number or viability (Figure S2a–c). IF of the ECM showed a decrease in the number of medin and CD63 foci and their size (Figure 1e). Quantification confirmed 3‐OMS treatment significantly decreased the number of CD63‐positive medin deposits per field of view. In addition, the size of the deposits was significantly reduced indicating sEVs could potentially be mediating medin aggregation (Figure 1e).

To corroborate the role of sEVs in medin deposition, knockdown of SMPD3 was also performed to decrease sEV secretion, confirmed by flow cytometry (Figure S2d). There was no effect of siSMPD3 treatment on cell number or viability (Figure S2e,f). Quantification of medin deposit size showed a significant decrease with siSMPD3 treatment compared with the control siRNA (Figure S2g).

To determine the proportion of sEV deposits associated with medin, the percentage of CD63 which colocalized with medin was quantified. Around 80% of CD63 deposits in the ECM colocalized with medin and this proportion was not significantly changed with 3‐OMS treatment, indicating 3‐OMS affects sEV secretion into the ECM and not medin loading on sEVs (Figure S2h). Western blotting of concentrated conditioned media showed no medin present when the media had been depleted of EVs by ultracentrifugation, with or without 3‐OMS treatment (Figure S2i), suggesting 3‐OMS treatment does not induce non‐EV associated medin secretion.

To investigate the role of sEVs on medin aggregation further, thioflavin T (ThT) assays were performed with recombinant monomeric medin with or without sEVs. Medin follows the classic amyloid aggregation profile with distinct lag, growth, and plateau phases (Figure 1f and Figure S2j). Nonlinear regression analysis with sigmoidal parameters was used to quantify the lag time of medin aggregation, which showed the exponential growth phase for medin alone started at between 105 and 115 min. The growth phase started at approximately 85 min for medin incubated with sEVs from VSMCs. Quantification showed sEVs significantly shortened the lag phase compared with medin alone, suggesting the association of medin with sEVs accelerates its aggregation.

### 2.2 VSMC senescence enhances medin accumulation through increased sEV secretion

**FIGURE 2:**
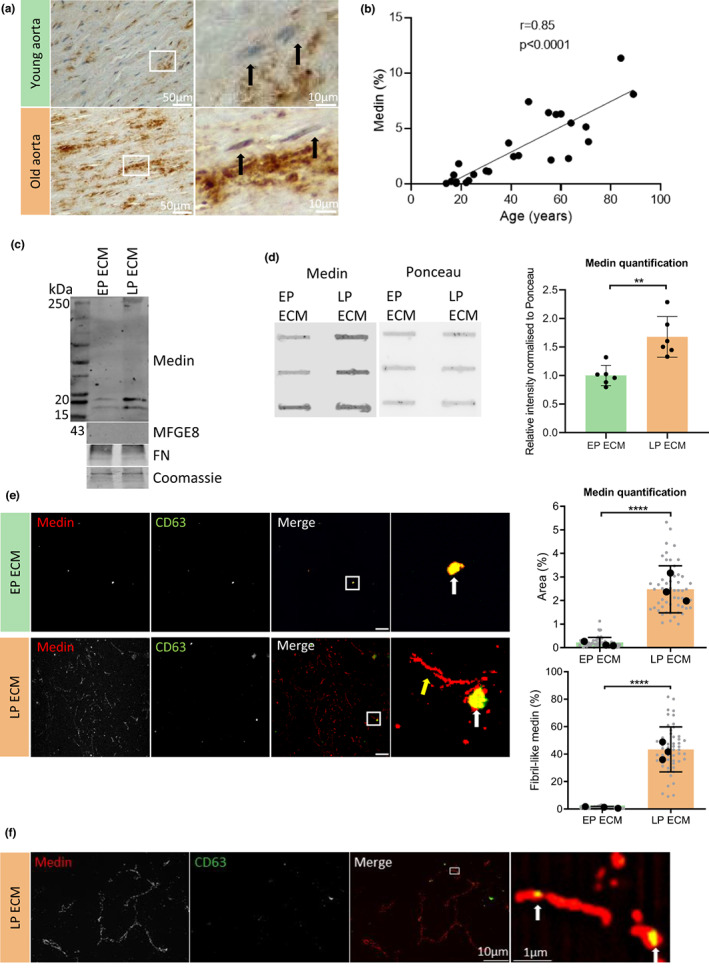
Vascular smooth muscle cell senescence enhances extracellular medin accumulation. (a) Immunohistochemistry of medin in the medial layer of aorta from human subjects. The black arrows highlight areas surrounding the nuclei which are not stained. (b) Correlation of medin staining with age (*n* = 25). Spearman correlation. (c) Representative Western blotting for medin and MFG‐E8 in early (EP) and late (LP) passage ECM. Fibronectin (FN) and Coomassie used as loading controls. (d) Slot blot for medin and quantification in EP ECM and LP ECM (*n* = 6 from 35‐year‐old female (35F), 22‐year‐old male (22 M), 20‐year‐old male (20 M)). Unpaired Student's t test, **p < 0.01. (e) Immunofluorescence of medin deposition in fibril‐like form (yellow arrow) in the LP ECM and colocalization with CD63 (white arrow) and quantification of medin deposition area and percentage of medin in fibril‐like form (*n* = 6 from 35F, 22 M, 20 M). Grey data points represent individual fields of view. Black data points represent average from each donor. Unpaired Student's t test, ****p < 0.001. Scale bar 25 μm. (f) Super resolution microscopy of medin in the LP ECM colocalized with CD63 (white arrows). All data is displayed as mean ± SD and was tested for normality using Shapiro–Wilk test.

To quantify the accumulation of medin in the vasculature with age, immunohistochemistry (IHC) was performed on aorta from human subjects from 14 to 89 years old. Medin deposition was sparse in the aortic medial layer of subjects under 40 years (Figure 2a,b). In older subjects, there was increased deposition of medin in the medial layer, which appeared predominantly extracellular, due to the absence of medin staining in cytoplasmic areas surrounding the nuclei. Quantification of the area of medin staining in the medial layer revealed a significant increase in medin accumulation in subjects over 40 years old with a strong, significant correlation between medin accumulation and age (Figure 2b).

This increased accumulation of medin with age led us to test whether senescence might impact on medin secretion and deposition. To do this, VSMCs were serially passaged in vitro until replicative senescence, which was verified using a senescence‐associated β‐galactosidase assay (Figure S3a). Late passage VSMCs also had increased expression of senescence markers, p16 and p21, and decreased expression of cell cycle regulator cyclin A2, compared to early passage VSMCs (Figure S3b–d). In addition, quantification of cell number 24 h following seeding showed a significant reduction in cell number, indicating reduced proliferation of late passage VSMCs (Figure S3e).

The decellularized ECM model was next used to observe changes in medin deposition in the ECM synthesized after 7 days from early and late passage VSMCs. Western blotting showed increased 15 and 20 kDa medin bands in the late passage ECM, with a larger band observed at approximately 250 kDa also detected (Figure 2c). There was no MFG‐E8 detected in the ECM by Western blotting. Slot blot and densitometric quantification confirmed there was an increase in total medin deposition in the late passage ECM, compared with the early passage ECM (Figure 2d). IF of the matrices revealed that medin was deposited in small, round punctate structures in the early passage ECM. In contrast, the late passage ECM contained increased medin deposition in long, fibril‐like structures with both structures showing co‐localization with CD63 (Figure 2e). Quantification of the staining showed a significant increase in medin area in the late passage ECM as well as increased deposition of the fibril‐like form of medin, indicated by a higher percentage of medin in fibril‐like form compared to the small round aggregate form. Super‐resolution microscopy showed these fibril‐like structures of medin formed around a core of CD63‐positive sEVs, resembling beads on a string (Figure 2f). The average length of individual fibril‐like structures was 7.8 ± 2.9 μm, while the average width was 180 ± 35 nm.

### 2.3 sEVs from senescent VSMCs accelerate medin aggregation

**FIGURE 3:**
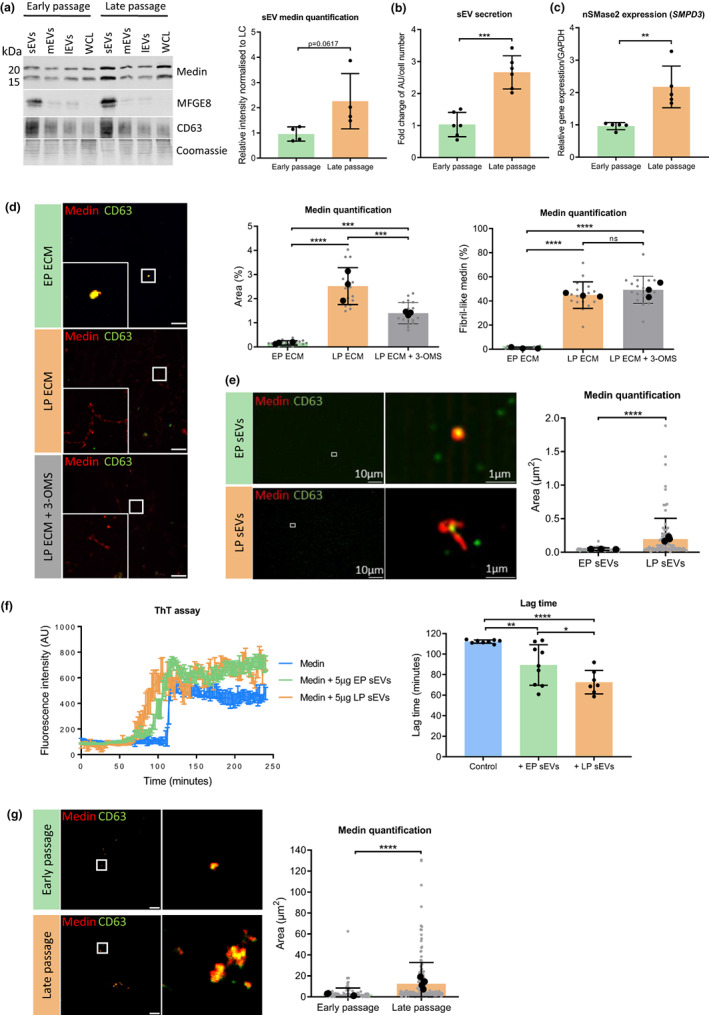
Vascular smooth muscle cell senescence increases medin deposition through increased sEV secretion. (a) Western blotting and quantification for medin in extracellular vesicles (EVs) from early and late passage VSMCs (*n* = 4–5 from 35‐year‐old female (35F)). CD63 used as a small EV (sEV) marker. Unpaired Student's t test. (b) Secretion of sEVs from early and late passage VSMCs, quantified by flow cytometry (*n* = 6 from 35F, 20‐year‐old male (20 M), 22‐year‐old male (22 M)). Unpaired Student's t test, ***p < 0.005. (c) RT‐qPCR of early passage and LP VSMCs showing gene expression of neutral sphingomyelinase (*SMPD3*, *n* = 5 from 35F). Unpaired Student's t test, **p < 0.01. (d) Immunofluorescence and quantification of early passage ECM (EP ECM) and late passage ECM (LP ECM) with or without EV secretion inhibitor, 3‐O‐methylsphingomyelin (3‐OMS). Grey data points represent a field of view and black data points represent experiment averages (*n* = 3 from 35F). One‐way ANOVA with Tukey post hoc test, ***p < 0.005, ****p < 0.001. (e) Super‐resolution microscopy of isolated EP and LP sEVs and quantification of the area of medin on the sEVs (*n* = 3 from 35F). Gray data points represent individual medin‐coated sEVs and black data points represent experiment averages. Unpaired Student's t test, ****p < 0.001. (f) Thioflavin T (ThT) assay and quantification of the aggregation of recombinant medin with EP and LP sEVs added (*n* = 6–9 from 35F). One‐way ANOVA with Tukey's post hoc test, *p < 0.05, **p < 0.01, ****p < 0.001. (g) Immunofluorescence and quantification of medin and CD63 deposition and colocalization in the ECM. Early and late passage VSMCs were seeded on EP ECM for 5 days before being lysed. Scale bar is 25 μm. Grey data points represent individual medin deposits and black data points represent experiment averages (*n* = 3 from 35F). Unpaired Student's t test, ****p < 0.001. All data is displayed as mean ± SD and was tested for normality using Shapiro–Wilk test.

To determine the mechanisms leading to increased medin secretion by senescent VSMCs, Western blotting was performed on EVs from early and late passage VSMCs, which showed increased secretion of both medin and MFG‐E8 in late passage sEVs (Figure 3a and Figure S4). EV secretion in was quantified by CD63‐beads assay and flow cytometry and this revealed late passage VSMCs secreted three‐fold more sEVs compared with early passage VSMCs (Figure 3b), suggesting increased sEV secretion and medin loading was causing the observed increases in medin deposition. Gene expression of sphingomyelin phosphodiesterase 3 (*SMPD3*), which regulates secretion of sEVs, was increased in late passage VSMCs compared with early passage VSMCs (Figure 3c).

To further investigate the role of senescent VSMC sEVs on medin aggregation, ECM was again synthesized by early and late passage VSMCs with or without 3‐OMS (Figure 3d). Quantification showed 3‐OMS significantly decreased the percentage area of medin in the late passage ECM; however, the ratio between fibril‐like and small, aggregate forms of medin was not affected suggesting additional mechanisms were promoting fibril formation in the late passage ECM (Figure 3d).

Next, super resolution microscopy was used to observe sEVs from early and late passage VSMCs. This showed late passage sEVs appeared to be coated with larger, fibril‐like medin aggregates compared with early passage sEVs (Figure 3e). This was confirmed by quantification of the area of each medin aggregate, suggesting late passage sEVs secrete medin in a more fibril‐like form or enhance aggregation on the sEV surface following secretion. To explore functional differences between early and late passage sEVs, ThT assays were performed with recombinant monomeric medin to examine effects on aggregation rates (Figure 3f). Quantification showed the late passage sEVs significantly shortened the lag phase of medin compared with early passage sEVs and medin alone, indicating a change in amyloid‐promoting potential of late passage sEVs. This was confirmed when early and late passage VSMCs were seeded on decellularized, early passage ECM. IF showed both the early and late passage VSMCs deposited small aggregates and not fibril‐like structures (Figure 3g), although there was a significant increase in aggregate size in the ECM when late passage VSMCs were seeded. These data suggest the secretion of late passage sEVs is not enough to trigger fibril formation in the early passage ECM, inferring additional effects of the late passage ECM are required for medin fibril formation.

### 2.4 VSMC senescence induces changes in sEV and ECM composition

To gain further insights into how senescence can promote medin aggregation, proteomics was performed on early and late passage VSMC‐derived sEVs and ECM. In total, 1694 proteins were identified in sEVs and 711 proteins in the ECM by mass spectrometry (Figure S5a). Four hundred and eighty‐seven of these proteins were common to both sEVs and ECM (Figure S5b) including sEV markers, amyloid precursors (including MFG‐E8), as well as collagens, proteoglycans and other ECM structural components and modifying enzymes, suggesting a complex interplay between sEV deposition and retention in the ECM with ECM production and remodeling.

**FIGURE 4:**
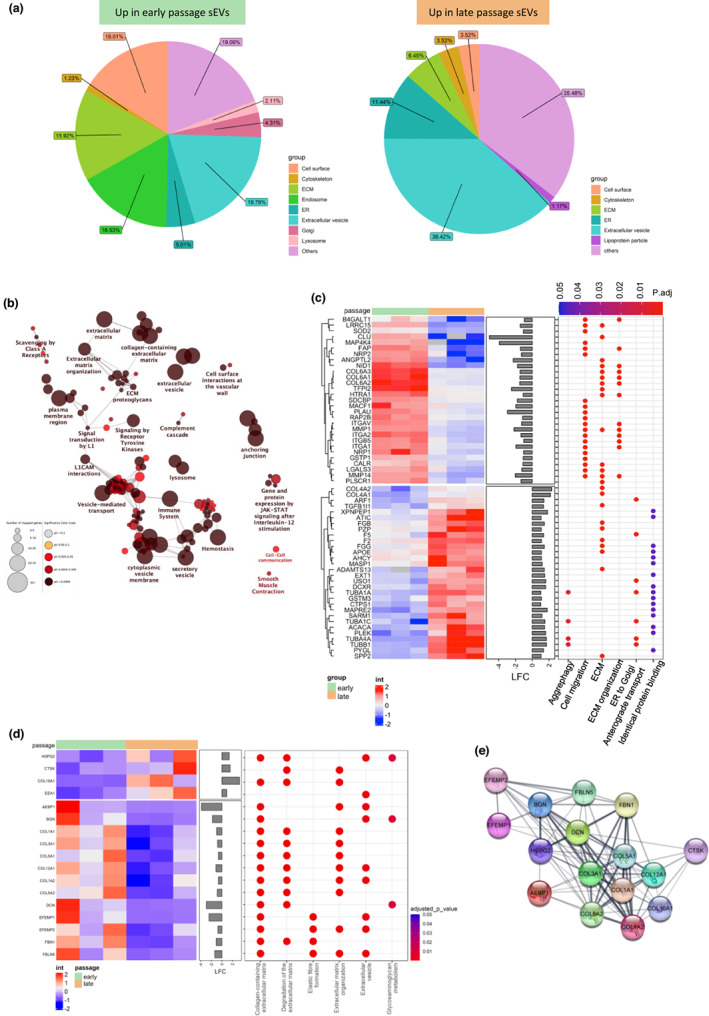
VSMC senescence induces changes in sEV and ECM composition. (a) Pie charts of subcellular localization of significantly changed sEV proteins which are upregulated in early passage (EP) sEVs or upregulated in late passage (LP) sEVs (*n* = 6 injections from 35‐year‐old female (35F) and 22‐year‐old male (22 M)). (b) Network of significantly changed proteins in LP sEVs showing the pathways which are affected. (c) Heatmap of LP vs EP sEVs with log2‐fold change (LFC) expression and enriched biological functions and pathways. (d) Heatmap of LP vs EP ECM differentially expressed proteins with LFC and enriched biological functions and pathways (*n* = 6 injections from 35F, 22 M, 20‐year‐old male (20 M)). (e) Protein–protein interaction network showing interactions between significantly upregulated and significantly downregulated ECM proteins in the LP ECM.

The most abundant proteins detected in both early passage and late passage EVs were COL6A3, COL6A1, FN1, ANXA2, VCAN, and HSPG2. However, comparison of the sEV protein abundances revealed 142 proteins were significantly increased in late passage sEVs and 267 proteins were significantly decreased, compared with early passage sEVs. As shown by Volcano plot (Figure S5c), the significantly changed proteins included ECM components such as COL6A1, COL6A2, COL6A3, MMPs, and clusterin which were decreased in late passage sEVs and COL4A1, COL4A2, FGB, FGG, and APOE which were increased. Comparisons of the ratio of significantly altered proteins from different cellular compartments between early passage and late passage sEVs (Figure 4a) revealed that in early passage sEVs, a larger proportion of proteins were categorized as ECM, endosomal, and cell surface components. These included 21 proteins involved in vesicle biogenesis and the endosomal sorting complexes required for transport (ESCRT) pathway, such as MVB12A, VPS28, VPS37B, SDCBP, and CHMP6, suggesting there are alterations in the EV biogenesis pathway between early and late passage VSMCs. This was consistent with a larger proportion of proteins that were ER‐ and EV‐related in late passage sEVs.

The protein interaction network of significantly altered proteins showed senescence induced significant changes in ECM organization, ECM proteoglycans, and vesicle‐mediated transport (Figure 4b). Pathway analysis performed on the significantly changed proteins revealed changes in ECM organization with several proteins involved in this pathway downregulated in late passage sEVs, suggesting a shift in ECM organization during senescence (Figure 4c). In addition, proteins involved in cell migration were significantly decreased in late passage sEVs. Significant pathways associated with upregulated proteins in late passage sEVs included endoplasmic reticulum (ER) to Golgi anterograde transport, identical protein binding and aggrephagy, suggesting changes in vesicle biogenesis and protein aggregation.

Proteomic analysis of early and late passage ECM revealed 38 proteins were significantly upregulated and 57 downregulated, shown by volcano plot (Figure S5d). Proteins decreased in the late passage ECM included ELN, FBN1, FBLN5, and proteoglycans, BGN and DCN. The upregulated proteins in the late passage ECM included COL10A1, HSPG2, the protease CTSK, and endosomal marker EEA1. Pathway analysis identified several significantly altered pathways involved in ECM remodeling, including ECM degradation, elastic fiber formation, and glycosaminoglycan (GAG) metabolism (Figure 4d). Importantly, protein interaction network analysis of the significantly changed ECM proteins showed several connections (Figure 4e).

### 2.5 Increased HSPG2 deposition with senescence enhances medin aggregation

**FIGURE 5:**
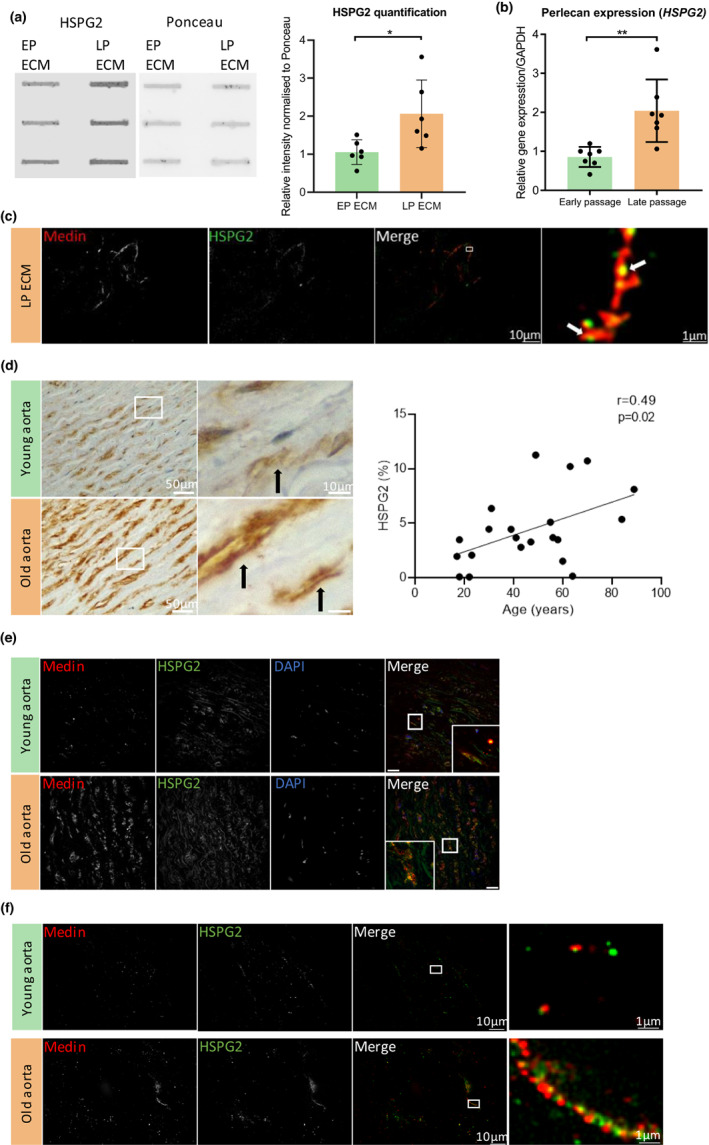
Medin and HSPG2 colocalize in the aortic medial layer and ECM. (a) Validation of the proteomics finding by slot blot and quantification of HSPG2 deposition in the early (EP) late passage (LP) ECM (*n* = 6 from 35‐year‐old female (35F), 20‐year‐old male (20 M) and 22‐year‐old male (22 M)). Unpaired Student's t test, *p < 0.05. (b) RT‐qPCR of HSPG2 gene expression in late passage VSMCs compared with early passage VSMCs (*n* = 7 from 35F, 20 M and 22 M). Unpaired Student's t test, **p < 0.01. (c) Super‐resolution microscopy of medin and HSPG2 colocalization in the LP ECM (white arrows). (d) Immunohistochemistry of extracellular HSPG2 in the aortic medial layer (black arrows). Quantification of the correlation of HSPG2 with age (*n* = 21). Spearman correlation. (e) Immunofluorescent staining of medin and HSPG2 deposition in human aortic medial layer. Scale bar 25 μm. (f) Super‐resolution microscopy of medin and HSPG2 colocalization in the human aorta sections. All data is displayed as mean ± SD and was tested for normality using Shapiro–Wilk test.

Given its increase in abundance in late passage ECM and its abundance in sEVs, we postulated that HSPG2 may enhance medin accumulation during senescence. Validation of the proteomics findings by slot blot and densitometric analysis confirmed there was significantly increased deposition of HSPG2 in the late passage ECM (Figure 5a). *HSPG2* gene expression was also significantly increased in late passage VSMCs (Figure 5b). IF staining of the late passage ECM showed fibril‐like medin was deposited associated with HSPG2 (Figure S6a) and super resolution microscopy confirmed colocalization of medin with HSPG2 in the late passage ECM with the fibril‐like medin forming around the HSPG2 deposits, again with a “beads on a string” pattern (Figure 5c). To determine whether these in vitro findings were relevant in vivo, IHC was used to examine the localization of HSPG2 in human aortas. HSPG2 was detectable in the aortic media of young subjects but deposition was increased in older subjects, and its deposition correlated significantly with age (Figure 5d). There was no significant correlation of HSPG2 and medin in the aorta of human subjects (Figure S6a). However, medin and HSPG2 were colocalized in human aortas of older subjects, shown by confocal (Figure 5e) and super resolution microscopy (Figure 5f). The HSPG2 was deposited in both punctate and fibrillar states and the medin colocalized with both forms in the ECM. Similar to the colocalization observed in the ECM in vitro, medin appeared to line up along the HSPG2 in a beaded pattern, forming fibril‐like structures.

**FIGURE 6:**
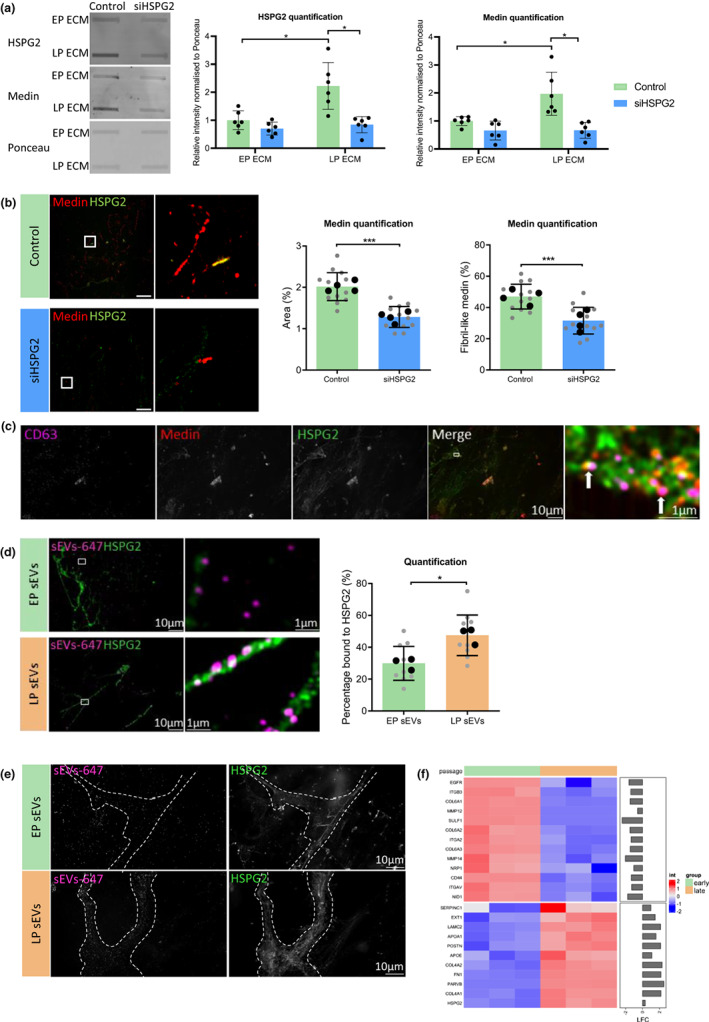
Increased HSPG2 deposition enhances medin aggregation. (a) Slot blot and quantification of medin and HSPG2 with HSPG2 siRNA treatment in the early passage (EP) and late passage (LP) ECM (*n* = 6 from 35‐year‐old female (35F)). Two‐way ANOVA with Tukey's post hoc test, *p < 0.05. (b) Immunofluorescence and quantification of medin area and fibril‐like form in LP ECM with control or siHSPG2 treatment. Gray data points represent individual fields of view and black data points represent experiment averages (*n* = 4 from 35F). Unpaired Student's t test, ***p < 0.005. (c) Super‐resolution microscopy of medin, CD63 and HSPG2 colocalization. (d) Immunofluorescence and quantification of sEVs from EP VSMCs (EP sEVs) or LP VSMCs (LP sEVs) binding to HSPG2 in decellularized ECM. Gray data points represent individual fields of view and black data points represent experiment averages (*n* = 3 from 35F). Unpaired Student's t test, *p < 0.05. (e) Super‐resolution microscopy of EP and LP sEVs bound to the ECM. Regions of HSPG2 deposition outlined in dashed white line. (f) Heatmap showing known HSPG2 binding proteins which were significantly changed in LP sEVs, compared to EP sEVs. All data are displayed as mean ± SD and were tested for normality using Shapiro–Wilk test.

To determine a functional role for HSPG2 in enhancing medin aggregation, ECM was synthesized by VSMCs treated with siRNA targeting *HSPG2*. Gene expression analysis validated the knock down of *HSPG2* in both early and late passage VSMCs (Figure S7a) and this was confirmed at the protein level by slot blot and densitometric analysis of the ECM (Figure 6a). HSPG2 knock down significantly decreased total medin and HSPG2 protein levels in the late passage ECM. siHSPG2 treatment did not significantly affect sEV secretion, cell viability or cell number (Figure S6b). In addition, HSPG2 knock down treatment of VSMCs did not affect the amount of medin in VSMCs, shown by Western blotting of VSMC lysates (Figure S6c). IF and quantification of the ECM with siHSPG2 also showed a significant decrease in medin deposition compared to the control ECM (Figure 6b). Importantly, there was also a significant decrease in the deposition of fibril‐like medin in the late passage ECM, indicating HSPG2 could be an enhancer of medin aggregation and also formation of fibril‐like structures. In contrast, siRNA knock down of COL10A1, which was also increased in the late passage ECM, had no significant effect on medin accumulation (Figure S7d, e).

The above data suggested that HSPG2 may play a role in vesicle capture into the ECM. Using super‐resolution microscopy, we confirmed colocalization of punctate CD63‐ and medin‐positive EVs coating the surface of HSPG2 fibers in the late passage ECM (Figure 6c). Next, to understand how early and late passage vesicles are captured and retained in the ECM, sEVs were isolated, fluorescently labelled then added onto the surface of early passage ECM in vitro. Super resolution microscopy showed that sEVs can bind to the ECM, however the binding pattern differed for early and late passage sEVs (Figure 6d). The late passage sEVs appeared to bind to HSPG2 fibrils and this was confirmed by quantification that showed a significant increase in the percentage of late passage sEVs colocalizing with HSPG2 compared to early passage sEVs. Early passage sEVs showed a diffuse pattern, binding evenly throughout the ECM, while late passage sEVs appeared to bind more exclusively in regions with HSPG2 deposition, suggesting a greater HSPG2 binding potential (Figure 6e). In support of this notion, proteomics identified changes in abundance of HSPG2 binding partners in sEVs, with a number significantly upregulated in late passage sEVs, including LAMC2, FN1, COL4A1, COL4A2, APOA1, and APOE (Figure 6f).

## 3 DISCUSSION

This study has elucidated for the first time how, during ageing, VSMCs regulate medin accumulation via secretion of sEVs, changes in sEV cargoes and ECM composition. We showed that VSMC senescence increased secretion of sEVs through upregulation of *SMPD3* and increased the accumulation of medin in the ECM by enhancing medin aggregation on the EV surface and EV binding to HSPG2 in the ECM. In addition, increased expression and deposition of HSPG2 in the senescent ECM triggered formation of fibril‐like medin structures, which could further enhance the development of AMA. Thus, targeting senescent VSMCs could be a potential therapeutic strategy to attenuate AMA formation.

### 3.1 sEVs mediate medin secretion, aggregation, and extracellular deposition

A study into the dynamics of medin amyloid assembly suggested a three‐stranded polymer of a medin fragment in β‐sheet formation is the basic unit from which amyloid fibrils are able to form (Gazit et al., 2007). The orientation of the monomers in ordered structures is mediated by specific side chain interactions, which contribute to stabilization of the three‐stranded medin aggregate. Consistent with this, we showed that VSMCs contained medin most likely in an aggregated form as only 15 and 20 kDa bands were observed by Western blot with two different antibodies. The absence of 5 kDa monomeric medin in EVs and VSMCs suggests medin rapidly oligomerizes into trimers or other small aggregates and does not remain in monomeric form in cells. In support of this, both soluble and insoluble forms of the medin precursor MFG‐E8 have been found in extracts from amyloid‐rich aortic media, however only insoluble medin was present, indicating medin occurs predominantly in aggregated form (Peng et al., 2005).

In AD, amyloidogenic cleavage of APP occurs predominantly in the endosomal pathway where acidic conditions promote amyloidogenic cleavage by β‐ and ϒ‐secretases, resulting in secretion of Aβ with sEVs (Niel, 2016; Perez‐Gonzalez et al., 2012). The medin precursor MFG‐E8 was highly enriched in sEVs and medin was deposited by CD63‐positive EVs in the ECM, highlighting a potential role of the endosomal pathway in MFG‐E8 cleavage into medin, although the mechanism and the proteases involved have yet to be elucidated. Cathepsin K (CTSK) was detected in the ECM in vitro and was significantly increased with senescence; however, a CTSK inhibitor had no effect on the production of medin in VSMCs (data not shown) indicating CTSK is not responsible for MFG‐E8 cleavage suggesting other as yet unidentified proteases are involved.

### 3.2 Senescence induces medin fibril formation through changes in sEV secretion

We demonstrated medin accumulates in the medial layer of the aorta and correlates very strongly with age. We showed that late passage VSMCs were senescent and had increased secretion and extracellular deposition of sEVs, leading to the deposition of fibril‐like medin in the ECM. Increased sEV release from senescent VSMCs correlated with increased expression of *SMPD3*, which catalyzes ceramide production, and blocking this pathway significantly reduced deposition of medin. Proteomics identified several proteins of the ESCRT pathway involved in the biogenesis of sEVs (Im et al., 2009; Juan & Furthauer, 2018), which were significantly decreased in sEVs from senescent VSMCs. These two changes potentially indicate a shift to ESCRT‐independent, SMPD3/ceramide‐dependent EV secretion by senescent VSMCs.

Medin was detected on the surface of sEVs. A ThT assay showed addition of sEVs accelerated medin aggregation by acting as a nucleus for formation of an amyloid seed, reducing the lag time and accelerating fibrillogenesis (Falker et al., 2016; Niel, 2016; Yuyama et al., 2008). Senescent VSMCs secreted more medin and sEVs from senescent VSMCs appeared to be coated in larger medin aggregates than those from early passage VSMCs. Moreover, these senescent sEVs shortened the lag phase of medin aggregation more than sEVs from early passage VSMCs, suggesting these aggregates were capable of further accelerating fibril formation. Proteomics also showed senescence induced changes in EV cargo, which could contribute to increased medin fibril formation on the EV surface; for example, increased APOE which has previously been colocalized with MFG‐E8 and amyloid in the aorta with ageing (Miura et al., 2019). In addition, APOE variants are a major risk factor for neurodegenerative diseases, such as AD and vascular dementia (Liu et al., 2012; Serrano‐Pozo et al., 2021). An alternate mechanism could be changes in lipid composition of sEVs from senescent cells (Buratta et al., 2017; Millner & Atilla‐Gokcumen, 2020), as previous studies have shown lipid membrane components can affect the rate of amyloid aggregation into fibrils (Brown & Bevan, 2017; Menon et al., 2020). Clearly, further work is required to investigate these mechanisms.

While previous studies have demonstrated cellular senescence causes changes in EV secretion and composition (Hitomi et al., 2020), EV secretion has also been shown to induce senescence in different cell types (Kadota et al., 2020; Xiao et al., 2021). Therefore, it is possible the increase in sEV secretion could promote VSMC senescence and cause a feedback loop with these processes enhancing each other.

### 3.3 Senescence‐associated HSPG2 deposition enhances medin aggregation and accumulation

While the above data implicated sEVs in the seeding and initial aggregation of medin in the ECM, a key observation was that blocking EV release or seeding senescent VSMCs on decellularized ECM did not influence formation of fibril‐like medin in the late passage ECM, indicating an additional trigger is required. Analysis of early and late passage ECM showed significant changes in the abundance of several ECM components with ageing. There was a significant decrease in proteins associated with elasticity, such as ELN, FBLN5, FBN1, and EFEMP1, suggesting cellular senescence induces the deposition of a less elastic ECM, which could contribute to age‐associated stiffening of the vessel wall (Thenappan et al., 2018). There was also a significant decrease in deposition of proteoglycans in the late passage ECM, including BGN and DCN, correlating with previous studies which have shown senescence‐associated decreases in expression of these proteoglycans in fibroblasts, intervertebral disc cells and chondrocytes (Dimozi et al., 2015; Takeda et al., 1992; Yudoh et al., 2005). The most significantly increased protein in the late passage ECM was COL10A1, suggesting a shift to a more chondrocyte‐like phenotype. However, the accumulation of COL10A1 in the ECM did not affect the deposition and aggregation of medin. In contrast, depletion of the proteoglycan HSPG2, which co‐localized with medin and was also increased in the late passage ECM, reduced the accumulation of medin and attenuated the formation of fibril‐like medin structures. Proteoglycans and GAGS have been shown to increase the rate of fibrillation of several proteins in vitro, including medin (Madine & Middleton, 2010). Therefore, the significant increase in *HSPG2* expression in senescent VSMCs, which correlated with its accumulation in the late passage ECM in vitro and aortic medial layer of old human subjects, strongly suggests age‐associated deposition of HSPG2 plays a role in enhancing medin fibrillation.

In addition to HSPG2 accumulating in the late passage ECM, HSPG2 was one of the most highly abundant proteins secreted in VSMC sEVs. HSPG2 plays a role in regulating ECM organization and stabilization through its interactions with a diverse range of ECM components (Guilak et al., 2021; Hayes et al., 2011). Colocalization was observed between sEVs and HSPG2 in the ECM and labelled sEVs from senescent VSMCs bound preferentially to HSPG2, suggesting it acted to template the fibril‐like medin structures in the ECM. Indeed, proteomics identified several significantly increased proteins in old sEVs that have been shown to interact with HSPG2, such as basement membrane components (FN1, BGN, LAMC2), collagens (COL1A1, COL1A2, COL3A1, COL4A1, COL4A2), and apolipoproteins (APOA1, APOE) implying senescence‐induced changes in sEV cargo enhance vesicle binding in the ECM. Further investigation is now required to understand the interactions that mediate vesicle adhesion and aggregation to promote medin fibril formation.

In this study, passaging of VSMCs to replicative senescence was used to mimic ageing. While there was a very strong association of VSMC senescence with EV secretion, changes in ECM composition and medin aggregation, there is a possibility these processes correlate with senescence and are not caused by senescence. Importantly, when seeding late passage VSMCs for experiments, the cells were already senescent (as shown by several different indicators of senescence). Therefore, it is reasonable to conclude that the differential effects on medin aggregation observed in these cells, compared with early passage cells, are due to senescence. To strengthen the evidence suggesting senescence is causative in medin accumulation during ageing, alternative methods of inducing senescence could be used and the effects of suppression of senescence studied.

The impact of medin aggregation in the vasculature is not completely understood, but previous research has shown acceleration of medin aggregation by heparin decreases the toxic effects of medin, suggesting formation of amyloid fibrils is a protective mechanism against the toxic nature of small aggregates (Madine & Middleton, 2010). In addition, studies have demonstrated medin aggregates induce pathological outcomes, such as endothelial dysfunction, vascular inflammation, and VSMC death (Migrino et al., 2017; Peng et al., 2007). However, the effect of pre‐fibrillar species and amyloid fibrils is complex, as research has shown amyloid deposits can contribute to reduced elasticity of vessels and weaking of the vessel wall (Davies et al., 2019; Pasha et al., 2020). Understanding of the pathological effects of medin aggregation is needed to identify potential therapeutic approaches to target or prevent AMA.

## 4 EXPERIMENTAL PROCEDURES

### 4.1 Cell culture and treatments

VSMCs were isolated from explants of human aortic tissues from three different donors: 35‐year‐old female (04‐35F‐11A), 20‐year‐old male (05‐20M‐18A), and 22‐year‐old male (05‐22M‐18A). All human materials were handled in compliance with the Human Tissue Act (2004, UK) and with ethical approval from the research ethics committee (REC reference: 13/LO/1950). Cells were cultured in M199 medium supplemented with 20% fetal bovine serum (FBS) and 1% penicillin–streptomycin‐glutamine (PSG) at 37°C with 5% CO_2_. To induce replicative senescence, VSMCs were passaged until their growth was arrested, which occurred between passages 20 and 25.

### 4.2 Vesicle isolation

EVs were isolated and purified by differential ultracentrifugation (UC). 24 h prior to isolation, VSMCs were washed twice with Earl's balanced salt solution (EBSS) and the medium changed to Dulbecco's modified eagle medium (DMEM) supplemented with 0.1% bovine serum albumin (BSA) and 1% PSG. The medium was collected and centrifuged at 1000 *g* to pellet large EVs (lEVs), followed by centrifugation at 10,000 *g* to pellet medium sized EVs (mEVs). Finally, small EVs (sEVs) were pelleted by UC at 100,000 *g*. All pellets were washed twice with phosphate buffered saline (PBS).

### 4.3 ECM synthesis

For synthesis of decellularized ECM, plates or glass coverslips were coated with gelatin, crosslinked with glutaraldehyde, which was then quenched with ethanolamine, as described previously (Franco‐Barraza et al., 2016). VSMCs were seeded at high confluency and cultured in media supplemented with 20% FBS, 1% PSG, and 50 μg/ml sodium L‐ascorbate for 7 days. To remove the VSMCs from the ECM, extraction buffer (0.1% Triton X‐100, 20 mM NH_4_OH in PBS) was added for 5 min at 37°C before washing three times in PBS to remove cell debris.

### 4.4 Immunoblotting

Extracellular matrix and cell extracts were prepared by washing in PBS and scraping in cold radioimmunoprecipitation assay (RIPA) buffer. Sample buffer (0.04% bromophenol blue, 8% SDS, 40% glycerol) with 50 mM dithiothreitol (DTT) was added before boiling for 5 min and separation by sodium dodecyl sulfate‐polyacrylamide gel electrophoresis (SDS‐PAGE). Proteins were transferred to PVDF membrane and blocked with 5% milk in PBS‐Tween. Primary antibodies were incubated with the membrane overnight at 4°C: mouse anti‐medin 18G1 (Prothena, 1:500), anti‐medin 6B3 (Prothena, 1:500), rabbit anti‐MFG‐E8 (Sigma, HPA002807, 1:500), mouse anti‐CD63 (BD Biosciences, 556,019, 1:1000), rabbit anti‐calnexin (CST, 2433 S, 1:1000), rabbit anti‐HSPG2 (Merck, SAB4301218, 1:500), mouse anti‐collagen type X (Merck, C7974, 1:500), and rabbit anti‐TSG101 (Abcam, ab125011, 1:1000). Secondary antibodies conjugated to IRDye 700CW were incubated for an hour before detection and quantification using an Odyssey imager and ImageStudio software: anti‐mouse IRDye 680RD (Li‐Cor, 925–68,070, 1:10000) and anti‐rabbit IRDye 800CW (Li‐Cor, 925–32,211, 1:10000).

Slot blotting was performed by applying samples to a Bio‐Dot SF Microfiltration Apparatus (Bio‐Rad) with a Welch Vacuum System (Model 2515). Proteins were blotted onto nitrocellulose membranes which were then stained with Ponceau S solution before blocking with 5% milk in PBS‐Tween and incubation with a primary antibody as before. To detect only surface proteins of EVs, the membranes were not washed or incubated with Tween‐containing buffers. Densitometric quantification of Western blotting and slot blotting was performed using Image Studio software and the values were normalized to a protein loading control.

A medin‐specific antibody (Prothena) was validated by transfection of U2OS cells. The region of the MFG‐E8 gene containing the medin fragment was cloned into a pcDNA3.1(+) mammalian expression vector and transfected into U2OS cells using FuGENE transfection reagent (Promega). The cell lysate was then collected for Western blotting.

### 4.5 Immunofluorescence and quantification

Decellularized ECM was fixed in 4% paraformaldehyde in PBS for 10 min followed by washing and blocking in 3% BSA in PBS for 1 h. Primary antibodies were added for 1 h before washing in PBS: mouse anti‐medin 18G1 (Prothena, 1:500), anti‐medin 6B3 (Prothena, 1:500), rabbit anti‐MFG‐E8 (Sigma, HPA002807, 1:500), rabbit anti‐fibronectin (Abcam, ab2413, 1:1000), rabbit anti‐CD63 (Santa Cruz, sc‐15,363, 1:500), rabbit anti‐annexin A6 (Abcam, ab31026, 1:250), rabbit anti‐fetuin A (pAS, 5359, 1:250), and rabbit anti‐HSPG2 (Merck, SAB4301218, 1:500). Fluorescent dye‐conjugated secondary antibodies were added for 1 h: AlexaFluor 488 anti‐rabbit (Molecular Probes, A21206, 1:500), AlexaFluor 546 anti‐mouse (Molecular Probes, A10036, 1:500). DAPI was used to stain nuclei for 2 min and following washing, the coverslips were mounted using Mowiol mounting medium. Z‐stacks of the ECM were imaged using a Nikon A1R confocal microscope with NIS‐Elements software. The area of extracellular medin or CD63 deposits was quantified using an automatic threshold method in ImageJ. For super‐resolution microscopy, the ECM was imaged using a Nikon iSIM with NIS‐Elements software.

Paraffin‐embedded tissue sections were deparaffinized, rehydrated, and heated in antigen retrieval solution. The sections were permeabilized using 0.1% Triton X‐100 in 0.2% gelatin for 20 min before blocking in 5% BSA in permeabilization solution. Primary antibodies were diluted in blocking buffer and incubated with the sections overnight at 4°C: mouse anti‐medin (Prothena, 1:1000), rabbit anti‐HSPG2 (Merck, SAB4301218, 1:500). Following a wash step in PBS, the secondary antibodies were diluted in blocking buffer and incubated for 1 h: AlexaFluor 488 anti‐rabbit (Molecular Probes, A21206, 1:500), AlexaFluor 546 anti‐mouse (Molecular Probes, A10036, 1:500). The nuclei were counterstained with DAPI for 5 min before dehydration, clearing in xylene and mounting using DPX mounting medium.

### 4.6 CD63‐beads assay and flow cytometry

Quantification of CD63 and CD81‐positive sEV secretion was performed by a CD63‐beads assay and flow cytometry analysis, adapted from a previously described protocol (Ostrowski et al., 2010). Anti‐human CD63 antibody (BD Biosciences, 556,019) was immobilized on 4 μm aldehyde‐sulfate beads and incubated with cell culture media overnight at 4°C. VSMCs were trypsinized and counted using a NC3000 NucleoCounter. The beads were washed with 2% BSA in PBS twice and incubated with anti‐CD81 antibody conjugated to phycoerythrin (BD Biosciences, 561957, 1:50), for 1 h at room temperature. The beads were washed twice, resuspended in PBS and analyzed by flow cytometry using a BD Accuri C6 flow cytometer. Arbitrary Units (AU) were calculated using FlowJo as mean fluorescence units multiplied by the percentage of positive beads and normalized to the cell number.

### 4.7 ExoView

Analysis of medin colocalization with sEVs was performed by ExoView. Briefly, sEVs were incubated with CD63 and CD9 antibody‐coated chips. Fluorescently labelled medin antibody was added to detect the medin‐positive sEVs captured by the antibodies. The chips were imaged automatically by ExoView R200 (NanoView Biosciences). Particle number was quantified based on the number of particles in an area of the chip, and all data were adjusted for dilution of the sample.

### 4.8 Thioflavin T assay and lag‐time quantification

To measure medin aggregation, 20 μM recombinant medin was added to 20 μM ThT in a 96‐well black, clear bottomed plate with or without 5 μM sEVs. The plate was incubated at 37°C for 4 h and gently agitated for 5 s before measurements were taken every 2 min with excitation at 440 nm in a fluorometer microplate reader. Quantification of lag time was performed by nonlinear regression with sigmoidal 4 parameters in GraphPad Prism, as previously described (Alvarez‐Martinez et al., 2011).

### 4.9 Senescence‐associated β‐galactosidase staining

Senescence‐associated β‐galactosidase assays (Cell Signaling Technology, 9860) were performed to confirm replicative senescence of late passage VSMCs using a commercial kit according to the manufacturer's instructions.

### 4.10 RNA isolation and quantitative reverse transcription PCR

RNA from VSMCs was collected using STAT60 and isolated using a phenol‐chloroform extraction method. cDNA was synthesized using Mu‐MLV reverse transcriptase with random and oligo primers, RNAse inhibitor, and dNTPs. qPCR was performed in triplicates using qPCRBIO SyGreen Mix and run in a StepOnePlus Real Time PCR system. PCR was performed in 20 μl reaction volumes at 95°C for 10 min followed by 40 cycles at 95°C for 5 s and 60°C for 1 min. Expression of target genes was calculated using the 2^‐ΔΔCt^ method, with *GAPDH* expression used for normalization.

### 4.11 Proteomics and bioinformatics analysis

Proteomics was performed on decellularized ECM and sEVs from early passage or senescent VSMCs. Briefly, the ECM was precipitated and deglycosylated with enzymes (chondroitinase, heparinase, keratinase, PNGase, O‐deglycosidase, and debranching enzymes) before denaturation using 6 M urea/2 M thiourea and DTT and alkylation with 50 mM iodoacetamide. Following precipitation and drying, the samples were resuspended in TEAB and digested with 0.2 μg/μl trypsin/Lys‐C overnight at 37°C. The samples were cleaned by loading onto C18 resin columns and resuspended in LC–MS buffer (2% acetonitrile, 0.05% TFA in water) before injection into a nanoflow liquid chromatography system (Dionex UltiMate 3000 RSLCnano). The eluate was then sprayed into a Q Exactive HF mass spectrometer using a MS2 setup.

Briefly, the sEVs were loaded onto a 10% Bis‐Tris gel and resolved, before being stained with Imperial protein stain. Following in‐gel trypsin digestion, samples were reduced with dithiothreitol and iodoacetamide and digested with trypsin overnight at 37°C. Peptides were separated by a nanoflow HPLC on an easy‐spray C18 nano column and analyzed by an Orbitrap Fusion Lumos mass spectrometer.

To account for differences during sample preparation, the abundance values were normalized to the total peptide amount of the most abundant sample. Proteome Discoverer software was used to search raw data files against human databases using Mascot. Proteomics data visualization was done using R studio and publicly available R packages from Bioconductor. For functional enrichment tests, the significant protein list was submitted to gProfiler web server to infer biological functions and pathways. Protein–protein interaction network was done in Cystoscape app 3.8.2 with attached STRING App.

### 4.12 Gene knockdown

For small interfering RNA‐mediated knockdown of HSPG2 and MFG‐E8, ON TARGETplus siRNAs or scrambled controls were transfected into VSMCs using HiPerfect transfection reagent for 2 days. The transfection mixture was replenished every 2 days for knockdown during 7‐day ECM synthesis.

### 4.13 Immunohistochemistry and quantification

Immunohistochemistry was performed on paraffin‐embedded human aortas. Aortic sections were deparaffinized, rehydrated before heat‐mediated antigen retrieval in sodium citrate buffer. Endogenous peroxidase activity was quenched, and non‐specific binding blocked with 10% serum before incubation with the primary antibodies: mouse anti‐medin (Prothena, 1:1000), rabbit anti‐HSPG2 (Merck, SAB4301218, 1:500). Biotinylated secondary antibodies were added, followed by amplification using avidin‐biotin complex (Vector Labs kits PK6101 and PK6102). Staining was developed with DAB peroxidase substrate and the sections counterstained with hematoxylin. The slides were mounted with DPX following dehydration. Images were taken on a Leica DM770 microscopy with LAS software. The area of medin and HSPG2 staining was quantified using ImageJ software with a threshold detection method and presented as a percentage of total area of the medial layer.

### 4.14 Statistical analysis

The results are presented as mean ± SD. All data were tested using Shapiro–Wilk normality test. An unpaired Student's t test was used to compare two independent groups. For comparison of multiple groups with one independent factor, a 1‐way ANOVA with Tukey's post hoc test was used. A 2‐way ANOVA with Tukey's post hoc test was used to analyze data with two independent factors. The results were described as statistically significant when *p* < 0.05. Data were obtained from a minimum of three independent experiments with a minimum of three technical replicates. Where stated, experiments were performed using VSMC isolates from three different donors.

## AUTHOR CONTRIBUTIONS

MW and CMS contributed to conception; JM and DM to resources; MW, DM, and CMS to experimental design; MW, SA, MM, and LS to acquisition of data; MW, SY, and SA to analysis and interpretation of data. MW and CMS wrote and revised the manuscript and all authors provided final approval of the submitted version.

## CONFLICT OF INTEREST

The authors declare that they have no conflicts of interest.

## DATA AVAILABILITY STATEMENT.

The mass spectrometry proteomics data supporting this study are available in Open Science Framework at osf.io/z25a6.

## Supporting information


Figure S1.

Figure S2.

Figure S3.

Figure S4.

Figure S5.

Figure S6.

Figure S7.

Table S1.
