# Supplementary figures and images for: Vascular smooth muscle cell senescence accelerates medin aggregation via small extracellular vesicle secretion and extracellular matrix reorganization
Source: Aging Cell. 2022 Nov 25;22(2):e13746. doi: 10.1111/acel.13746 (PMC9924949; doi:10.1111/acel.13746)

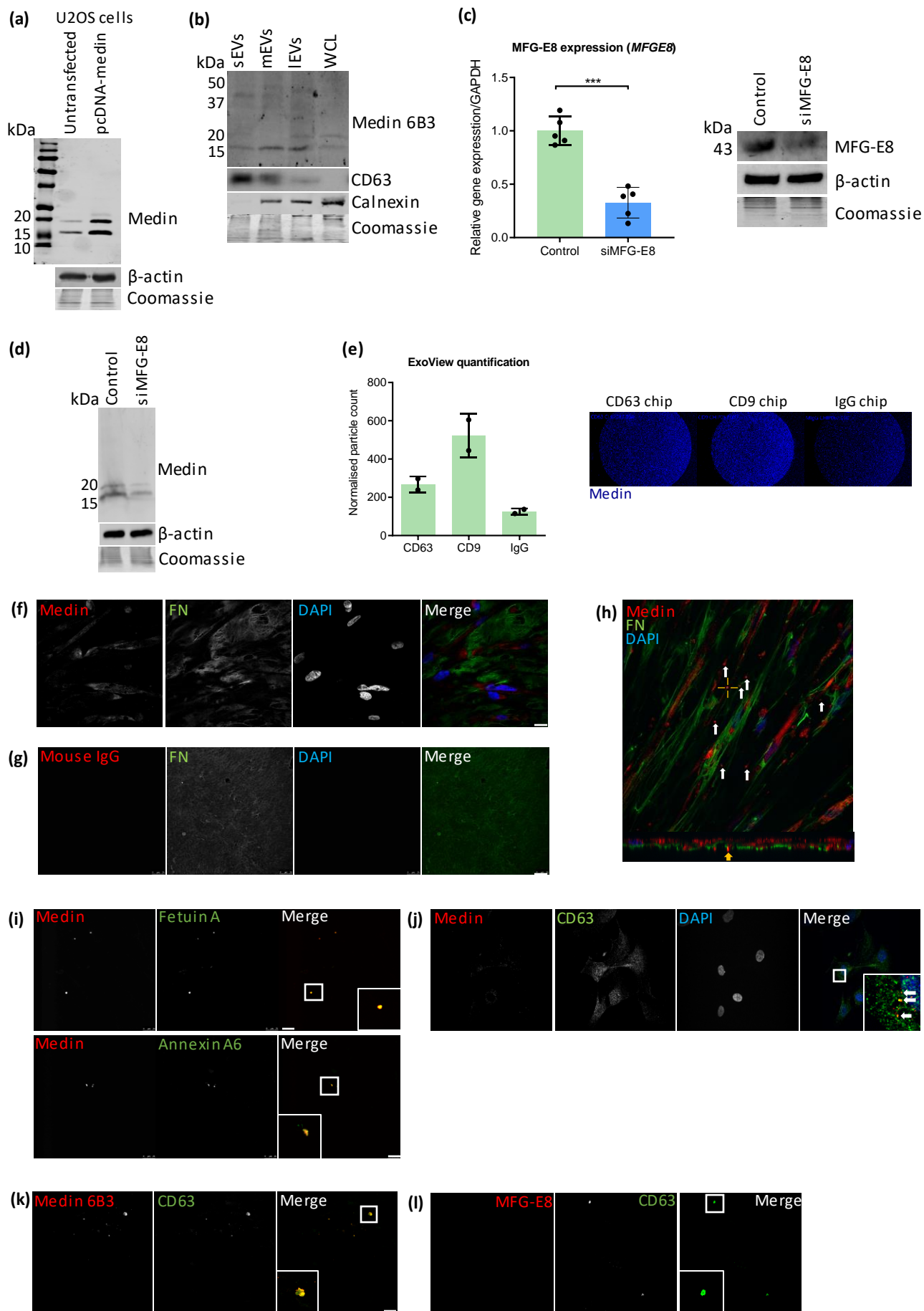

Supplement: Supplementary file 1 — Figure S1. [file ACEL-22-e13746-s008.pdf]

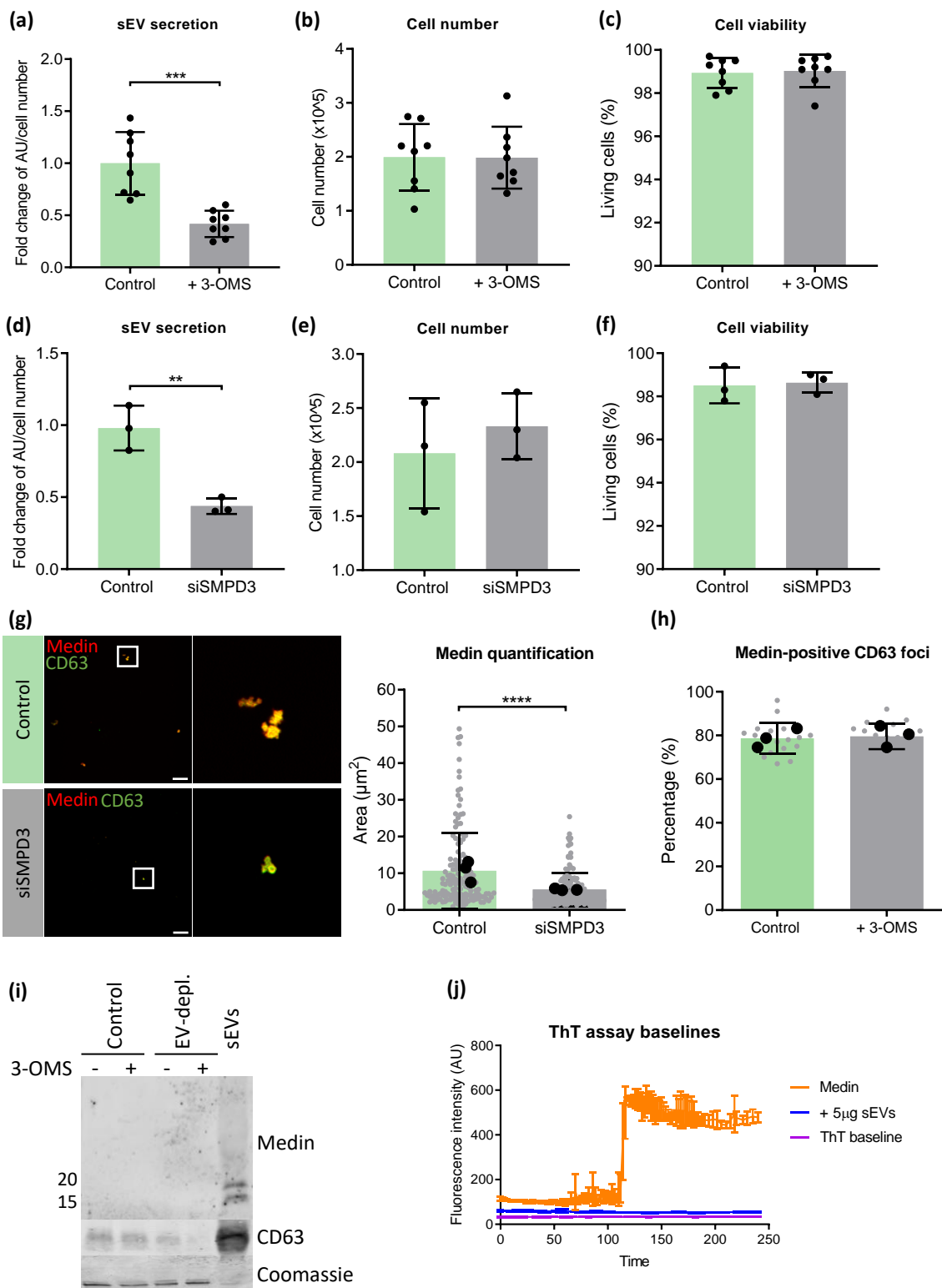

Supplement: Supplementary file 2 — Figure S2. [file ACEL-22-e13746-s007.pdf]

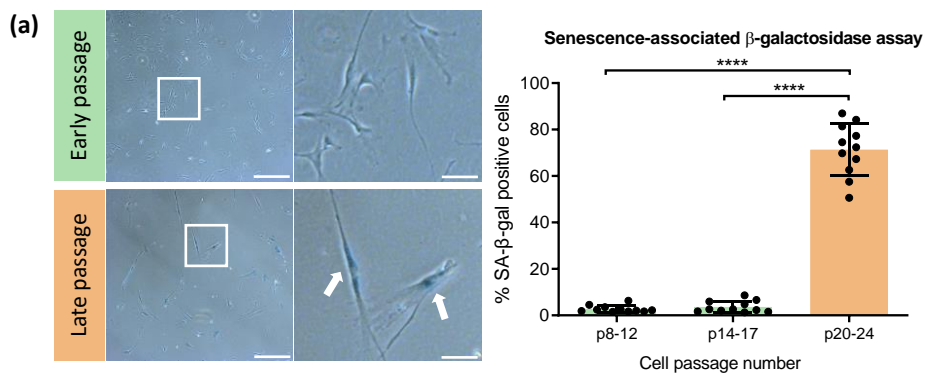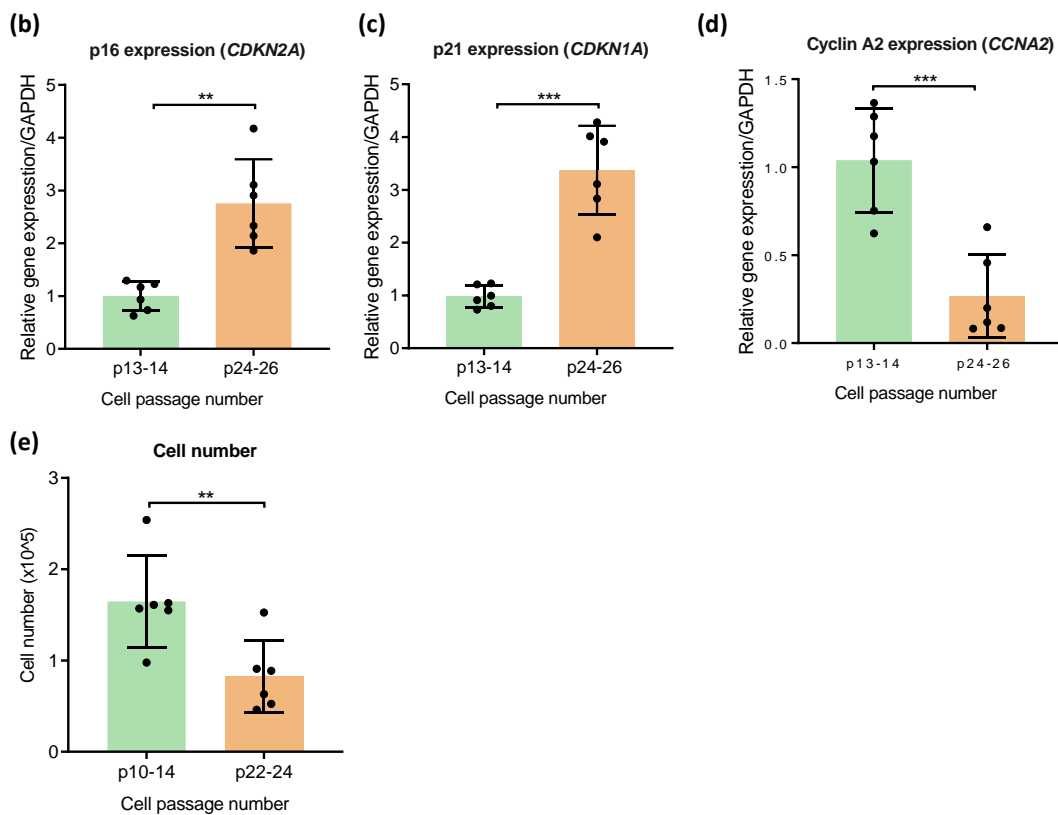

Supplement: Supplementary file 3 — Figure S3. [file ACEL-22-e13746-s003.pdf]

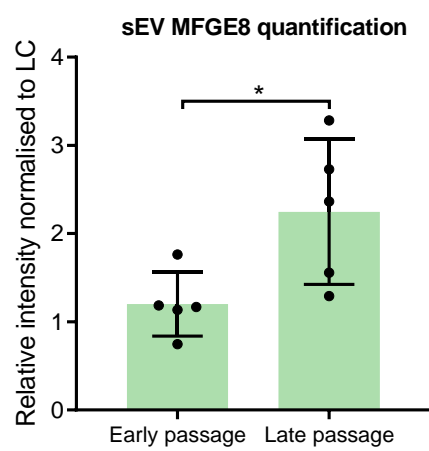

Supplement: Supplementary file 4 — Figure S4. [file ACEL-22-e13746-s005.pdf]

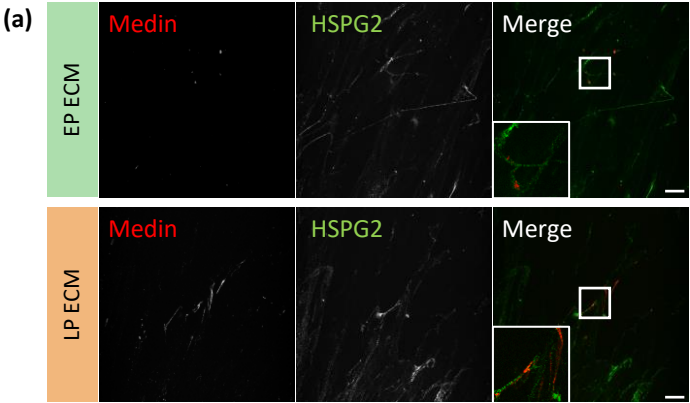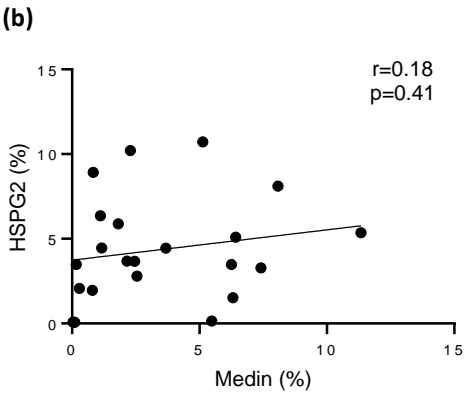

Supplement: Supplementary file 6 — Figure S6. [file ACEL-22-e13746-s001.pdf]

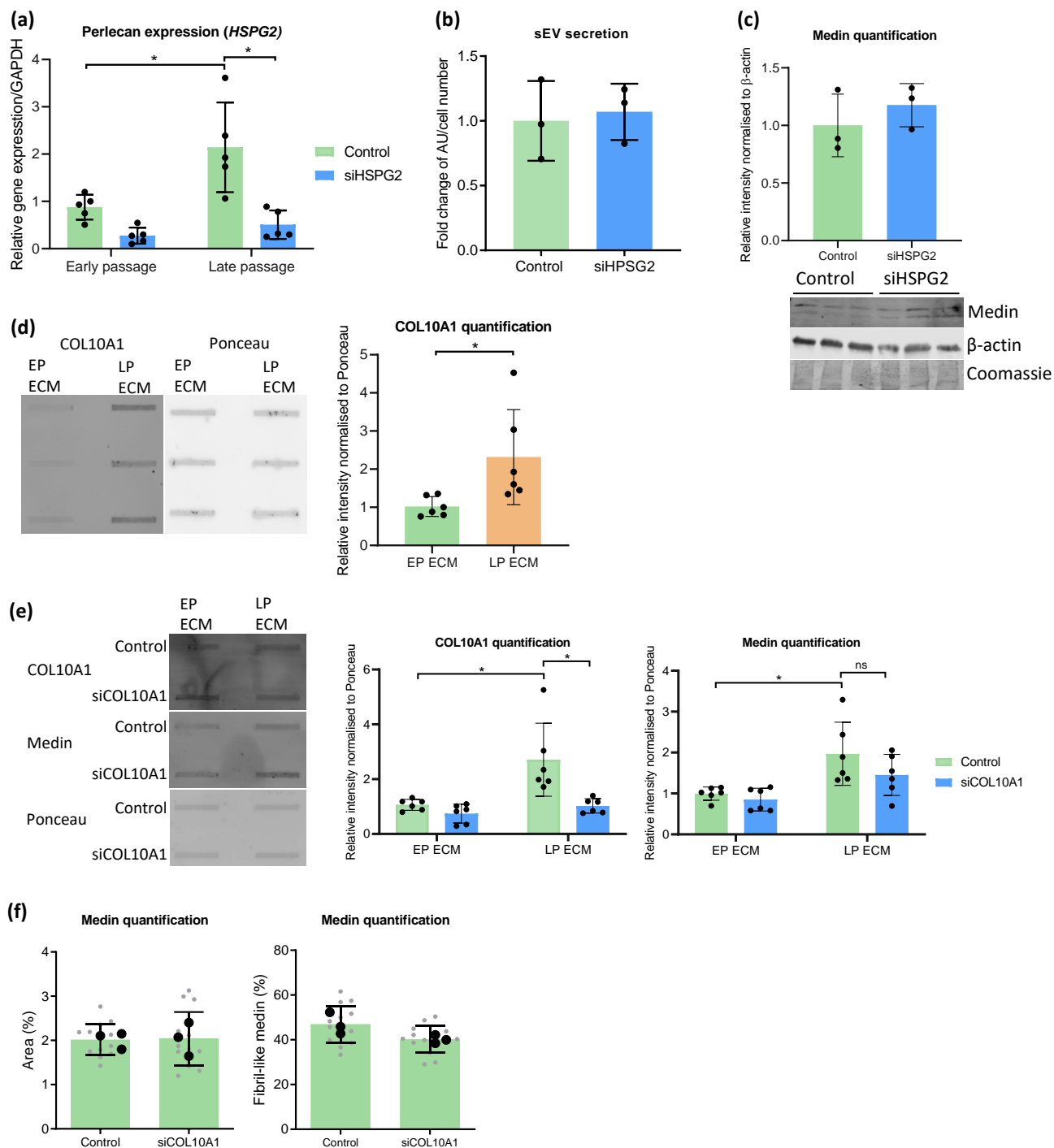

Supplement: Supplementary file 7 — Figure S7. [file ACEL-22-e13746-s004.pdf]
