# Supplementary material for: Vascular smooth muscle cell senescence accelerates medin aggregation via small extracellular vesicle secretion and extracellular matrix reorganization
Source: Aging Cell. 2022 Nov 25;22(2):e13746. doi: 10.1111/acel.13746 (PMC9924949; doi:10.1111/acel.13746)
Supplement: Supplementary file 5 — Figure S5. [file ACEL-22-e13746-s002.pdf]

(a)

Component	# Proteins identified	# Proteins changed
sEVs	1694	142↑ 267↓
ECM	711	38↑ 57↓

↑ Significantly increased in LP, q<0.05, LFC>0.5

↓ Significantly decreased in LP, q<0.05, LFC<-0.5

(b)

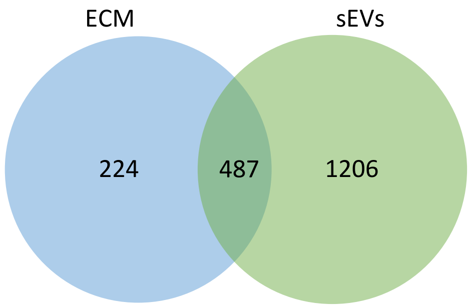

sEV markers	Amyloid precursors	Collagens	ECM modifying enzymes	Proteoglycans	Structural ECM proteins
TSG101	MFGE8	COL1A1 ↑	MMP1 ↓	HSPG2 ↑	FN1 ↑
CD81	APP	COL1A2 ↑	MMP2 ↓	AGRN	LAMB1
CD63 ↓	SOD1	COL3A1 ↑	MMP14 ↓	VCAN	LAMA4
SDCDP	GSN	COL4A1 ↑	TIMP3	BGN ↑↓	ECM1
ALIX		COL4A2 ↑	ADAMTSL1 ↑	HAPLN1	EMILIN1
FLOT1		COL6A1 ↓	LOX		FBLN1
FLOT2		COL6A2 ↓	LOXL1		FBLN2
		COL6A3 ↓	LOXL2		FBN1 ↓
		COL12A1 ↓	PLOD1		NID2
		COL16A1 ↑	PLOD2		EFEMP1 ↓↓
					FGG ↑
					FGB ↑
					TNC
					MATN2
					POSTN ↑
					VTN

↑ Significantly increased in LP sEVs ↑ Significantly increased in LP ECM

↓ Significantly decreased in LP sEVs ↓ Significantly decreased in LP ECM

(c)

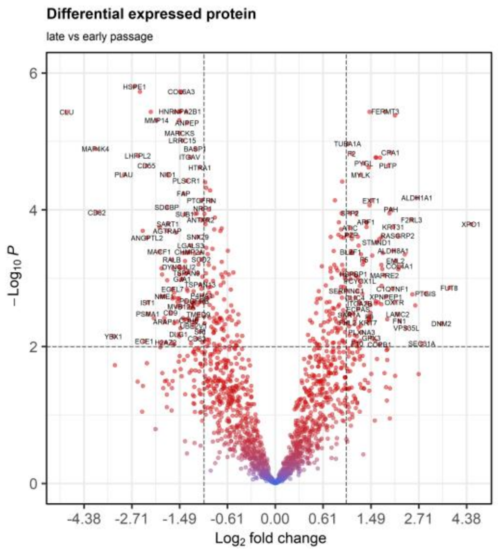

Log$_2$ fold change cutoff, 1; p.adj.value cutoff, 0.01

(d)

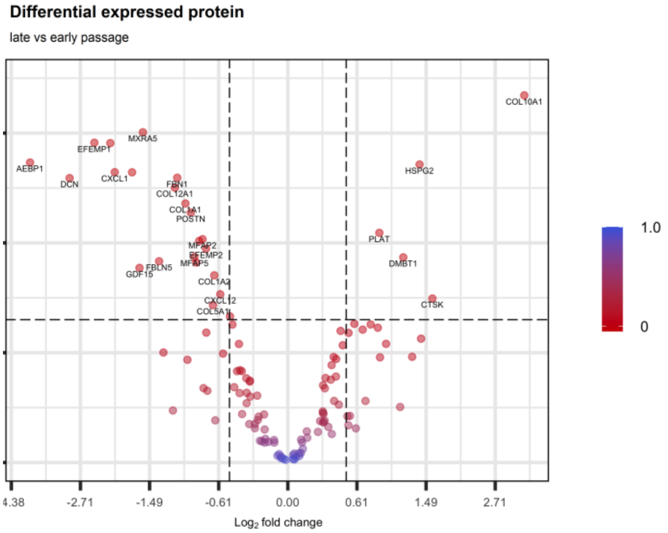

Log$_2$ fold change cutoff, 0.5; p.value cutoff, 0.05
