# Supplementary material for: Vascular smooth muscle cell senescence accelerates medin aggregation via small extracellular vesicle secretion and extracellular matrix reorganization
Source: Aging Cell. 2022 Nov 25;22(2):e13746. doi: 10.1111/acel.13746 (PMC9924949; doi:10.1111/acel.13746)
Supplement: Supplementary file 8 — Table S1. [file ACEL-22-e13746-s006.docx]

**
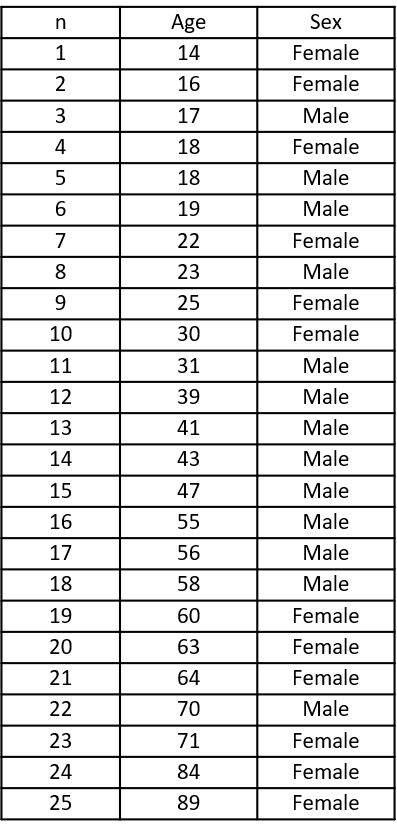
**

**Supplementary Table 1**: Aorta samples used for immunohistochemistry

**Supplementary figure legends**

**Supplementary Figure 1:** (a) Western blotting of cell lysates from U2OS cells with or without transfection of a vector containing the medin fragment of MFG-E8. β-actin and Coomassie were used as loading controls. (b) Representative Western blot of small (sEVs), medium (mEVs), large (lEVs) and whole cell lysate (WCL) for medin with 6B3 antibody. CD63, calnexin and Coomassie were used as controls. (c) RT-qPCR and representative Western blot of control and MFG-E8 siRNA treated VSMCs (n=5 from 35F). Mean ± SD, unpaired Student’s t-test, ***p<0.005. (d) Representative Western blot of control and siMFG-E8 treated VSMCs for medin. β-actin and Coomassie were used as loading controls. (e) Fluorescent imaging and quantification of medin positive sEVs (blue) captured on CD63, CD9 or control IgG antibody-coated chips (n=2 from 35-year-old female (35F)). The particle count was quantified as the number of vesicles in a defined area of the antibody chip. Mean ± SD. (f) Immunofluorescent staining (IF) of VSMCs prior to lysis following ECM synthesis, showing deposition of ECM with fibronectin (FN). (g) IF of decellularised ECM, showing deposition of an intact matrix with FN, the absence of cells with no DAPI staining and no background staining with mouse control IgG antibody. (h) Z-stack of ECM showing medin deposition in the ECM before VSMC lysis, indicated by arrows. (i) IF of decellularised ECM showing colocalisation of medin and EV markers, Fetuin A and Annexin A6. (j) IF of VSMCs for medin, CD63 and DAPI. (k) IF of ECM for medin with the 6B3 antibody clone and colocalisation with CD63. (l) IF of ECM for MFG-E8 and CD63. Scale bars are 25μm.

**Supplementary Figure 2:** (a) Flow cytometry analysis of sEV secretion from VSMCs treated with EV inhibitor, 3-o-methylsphingomyelin (3-OMS, n=8 from 35-year-old female (35F)). Mean ± SD, unpaired Student’s t-test, ***p<0.005. Analysis of (b) cell number and (c) cell viability with 3-OMS treatment (n=8 from 35F). (d) Flow cytometry of sEV secretion with siSMPD3 treatment (n=3 from 35F). Mean ± SD, unpaired Student’s t-test, **p<0.01. Effect of siSMPD3 on (e) cell number or (f) cell viability. (g) Immunofluorescent staining and quantification of ECM treated with control or SMPD3 siRNA. Grey data points represent individual medin deposits and black data points represent experiment averages (n=3 from 35F). Mean ± SD, unpaired Student’s t-test, ***p<0.005. (h) Quantification of percentage of CD63 foci colocalised with medin in the ECM (n=3 from 35F). Mean ± SD. (i) Western blotting of medin and CD63 in sEVs and control or EV-depleted conditioned media with or without 3-OMS treatment. Coomassie was used as a loading control. (j) Thioflavin T (ThT) assay of recombinant medin peptide showing aggregation into amyloid fibrils after around 120 minutes. Baseline readings were taken for sEVs and ThT alone (n=8 from 35F). Mean ± SD.

**Supplementary Figure 3:** (a) Senescence-associated β-galactosidase (SA-β-gal) assay used to validate the accumulation of senescent VSMCs at late passages. Senescence was detected by blue staining in VSMCs (white arrows). (n=11 from 35-year-old female (35F), 20-year-old male (20M) and 22-year-old male (22M)). Mean ± SD, one-way ANOVA with Tukey’s post hoc test, ****p<0.001. RT-qPCR of early passage and late passage VSMCs showing expression of senescence markers (b) p16, (c) p21 and (d) cyclin A2 (n=6 from 35F). Mean ± SD, unpaired Student’s t-test, **p<0.01, ***p<0.005. (e) Quantification of cell number of VSMCs at late passages compared to early passages (n=6 from 35F, 20M and 22M). Mean ± SD, unpaired Student’s t-test, **p<0.01.

**Supplementary Figure 4:** Quantification of Western blot (shown in Figure 3A) of extracellular vesicles (EVs) from early passage and late passage VSMCs (n=5 from 35F). Mean ± SD, unpaired Student’s t-test, *p<0.05.

**Supplementary Figure 5:** (a) Table showing the proteomic analysis of sEVs and ECM from early passage VSMCs (EP sEVs) and from late passage VSMCs (LP sEVs) showing numbers of proteins significantly increased or decreased (n=6 injections from 35-year-old female (35F), 22-year-old male (22M) and 20-year-old male (20M)). (b) Venn diagram showing the proteins detected in both ECM and sEVs. There were 487 proteins common in both data sets. The table lists some proteins of interest and denotes whether they are significantly increased or decreased in LP sEVs or ECM. Volcano plots showing differentially expressed proteins in (c) EP and LP sEVs and (d) ECM.

**Supplementary Figure 6:** (a) Immunofluorescent staining of medin and HSPG2 in decellularised ECM from early passage VSMCs (EP ECM) and late passage VSMCs (LP ECM). Scale bar is 25μm. (b) Correlation of medin and HSPG2 quantification (as percentage area). Spearman correlation.

**Supplementary Figure 7:** (a) RT-qPCR of early passage and late passage VMSCs treated with control or HSPG2 siRNA (n=5 from 35-year-old female (35F)). Mean ± SD, two-way ANOVA with Tukey’s post hoc test, *p<0.05. (b) Flow cytometry analysis of sEV secretion with siHSPG2 treatment (n=3 from 35F). (c) Western blotting and quantification of medin in cell lysates from control or siHSPG2 treated VSMCs. (n=3 from 35F). (d) Slot blot and quantification showing deposition of COL10A1 in ECM from late passage VSMCs (LP ECM, n=6 from 35F, 22-year-old male (22M) and 20-year-old male (20M)). Mean SD, unpaired Student’s t-test, *p<0.05. (e) Slot blot and quantification showing effect of siCOL10A1 on deposition of COL10A1 and medin in the LP ECM (n=6 from 35F). Mean SD, two-way ANOVA with Tukey’s post hoc test, *p<0.05. (f) Quantification of immunofluorescent staining of medin area or medin fibril-like formation with siCOL10A1. Grey data points represent individual fields of view and black data points represent experiment averages (n=3 from 35F). Mean ± SD, unpaired Student’s t-test.
